# Inflammatory Immune Responses Trigger Rejection of Allogeneic Fibroblasts Transplanted into Mouse Skin

**DOI:** 10.1177/09636897221113803

**Published:** 2022-07-30

**Authors:** Ali Farrokhi, MohammadReza Rahavi, Sumin Jo, Reza Jalili, C. James Lim, Aziz Ghahsary, Gregor S. D. Reid

**Affiliations:** 1 Michael Cuccione Childhood Cancer Research Program, BC Children’s Hospital Research Institute, Vancouver, BC, Canada; 2 Department of Pediatrics, The University of British Columbia, Vancouver, BC, Canada; 3 Burn & Wound Healing Research Group, Division of Plastic Surgery, Department of Surgery and International Collaboration on Repair Discoveries, The University of British Columbia, Vancouver, BC, Canada

**Keywords:** fibroblasts, wound healing, allogeneic cell transplantation, immunosuppression

## Abstract

Fibroblasts, or their homolog stromal cells, are present in most tissues and play an essential role in tissue homeostasis and regeneration. As a result, fibroblast-based strategies have been widely employed in tissue engineering. However, while considered to have immunosuppressive properties, the survival and functionality of allogeneic fibroblasts after transplantation remain controversial. Here, we evaluated innate and adaptive immune responses against allogeneic fibroblasts following intradermal injection into different immune-deficient mouse strains. While allogeneic fibroblasts were rejected 1 week after transplantation in immunocompetent mice, rejection did not occur in immunodeficient γ chain–deficient NOD-SCID (NSG) mice. T-cell- and B-cell-deficient RAG1 knockout mice showed greater loss of fibroblasts by day 5 after transplantation compared with NSG mice (*P* ≤ 0.05) but prolonged persistence compared with wild-type recipient (*P* ≤ 0.005). Loss of fibroblasts correlated with the expression of proinflammatory chemokine genes and infiltration of myeloid cells in the transplantation site. Depletion of macrophages and neutrophils delayed rejection, revealing the role of innate immune cells in an early elimination of fibroblasts that is followed by T-cell-mediated rejection in the second week. These findings indicate that the application of allogeneic fibroblasts in tissue engineering products requires further improvements to overcome cell rejection by innate and adaptive immune cells.

## Introduction

Mammalian tissues are composed of a variety of different cell types, but fibroblasts or their stromal cell counterparts are common to almost all tissues^1^. Fibroblasts accelerate extracellular matrix remodeling and provide a microenvironment to support the migration, proliferation, and differentiation of other cells^2^. Fibroblasts also produce cytokines and growth factors that have regulatory effects on many other cells, including epidermal, vascular, and lymphatic endothelial cells^2–4^, which contribute to their critical roles in tissue homeostasis, tissue repair, wound healing, inflammation, and fibrosis^5,6^. Given these multiple functions, fibroblast-based strategies have been applied in the engineering of a broad range of different tissues including skin^7^, heart^8,9^, liver^10,11^, and musculoskeletal tissue, such as tendon^12,13^, ligament^14^, bone^15^, cartilage^16^, and muscle^17^. Currently, fibroblast-based products for skin regeneration are commercially available or in clinical trials^18^.

Fibroblasts used in tissue-engineered products can be either autologous or allogeneic. In addition to being less expensive to produce^19^, allogeneic fibroblasts offer several advantages, including reduced morbidity at donor sites and avoidance of treatment delays resulting from the time needed for autologous cell isolation and culture^20^. Furthermore, in chronic diseases like diabetes, autologous fibroblasts have been altered due to extrinsic factors and are defective in extracellular matrix production and wound healing capacities^21,22^. However, the viability and immunogenicity of allogeneic cells after transplantation into an immunocompetent host have been controversial, and the usefulness of transplanted allogeneic fibroblasts is still unclear^23–29^. One possible explanation for this controversy could be that fibroblasts, as sentinel cells, have immunomodulatory roles that may be proinflammatory^30–34^ or immunosuppressive^35–40^ depending on the microenvironment conditions. The immunosuppressive capacity of fibroblasts has been studied mostly *in vitro*; however, skin fibroblasts can suppress inflammation *in vivo* in a model of arthritis^41^, and cancer-associated fibroblasts (CAFs) can exert immune suppression in the tumor microenvironment^42–44^.

As immunosuppressive properties could be harnessed to enhance the survival of allogeneic fibroblasts in tissue engineering settings, we first investigated the fate of repopulating allogeneic fibroblasts within an acellular dermal matrix (ADM) applied as wound coverage in a full-thickness skin wound model in mice. Upon observing no benefit for wound healing by recellularizing ADM, we tracked the viability of fibroblasts transplanted via intradermal injection into a range of immune-compromised murine strains. Our results reveal a prominent role for innate immune cells in the early rejection of allogeneic fibroblasts in the first 5 days after transplantation, followed by a T-cell-mediated response in the second week. These findings support the previous reports that potential immunomodulatory properties do not protect transplanted fibroblasts from classical allogeneic immune responses^24,25,27^ and provide a mechanistic explanation for this outcome.

## Materials and Methods

### Ethics Statement

All animal experiments were performed in accordance with the Canadian Council of Animal Care and a University of British Columbia Animal Care Committee–approved protocols (A15-0187 and A18-0366).

### Mice

Male and female C57BL/6 mice, aged 3 to 4 months, were used as skin donors to culture fibroblasts for transplantation studies. Recipients were 2 to 4 months old female and male wild-type (wt) BALB/c, recombination-activating genes (RAG)-deficient (RAG1−/−), CD1-deficient (CD1−/−), Ja18-deficient (JA18−/−), Stat4-deficient, Stat6-deficient BALB/c mice and common γ chain–deficient NOD-SCID (NSG) mice. All mice were obtained from The Jackson Laboratory (Bar Harbor, Maine, USA) and maintained as in-house colonies under specific pathogen-free conditions.

### Dermal Fibroblast Attachment and Viability on ADMs Scaffolds

Mouse dermal fibroblasts from C57BL/6 mice were seeded onto ADM from wt-BALB/c at a density of 100 × 10^3^ and maintained in culture. Cell adherence and viability was assessed 24 hours after seeding, using a Live/Dead toxicity assay (Molecular Probes^®^, Invitrogen, Carlsbad, California, USA) followed by visualization by confocal microscopy. To perform the assay, the ADMs containing the cells were washed three times with phosphate-buffered saline (PBS) and incubated with a mixture of ethidium homodimer and calcein-AM according to the manufacturer’s instructions. After 30 minutes, the scaffolds were washed three times with PBS and visualized using a Zeiss AxioObserver Z1 confocal microscope fitted with a CSU-X1 spinning disk (Yokogawa Electric, Tokyo, Japan) and AxioVision 4.8 (Carl Zeiss microscopy, NY, USA), and the images were analyzed by Zen software. For long-term viability assessments, tissue culture medium was assessed 24 hours after seeding the cells and then every 3 days for the 3-week culture, for the presence of lactate dehydrogenase (LDH) (Pierce™ LDH Cytotoxicity Assay Kit), according to the manufacturer’s instructions. Lysate from serially diluted *in vitro* cultured fibroblasts (0.5–20 × 10^3^ cells) used as an assay control to detect LDH.

### Transplantation Procedure for Full-Thickness Wound Treatment

**Figure 1.**
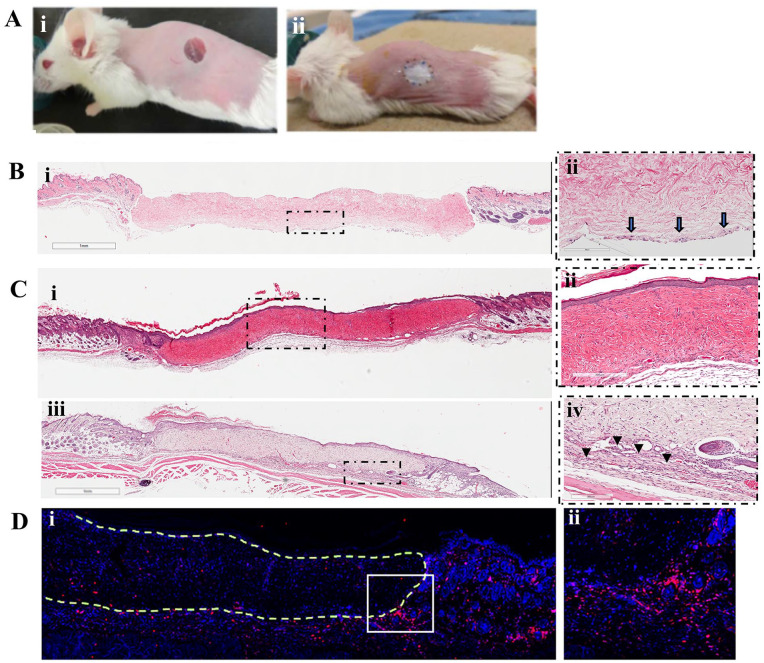
Full-thickness wounds and transplantation of ADM or recellularized ADM (Ai, ii). The presence of fibroblasts seeded on ADM was confirmed by analyzing recellularized ADM graft, 1 hour after transplantation. Arrows indicate cells attached to the surface of the ADM facing the wound bed (B.i, ii). Three weeks after transplantation, ADM-transplanted wound (C.i, ii) and recellularized ADM–transplanted wound (C.iii, iv) show engraftment to surrounding host tissue with complete epithelialization. However, in recellularized ADM group, high cellularity can be seen at the interface of graft/host tissue (arrow heads) (C.iv). Staining of the graft for pan-leukocyte marker, CD45, revealed that majority of the cells in the high-cellularity regions are CD45+ cells. (D.i, ii). Figures are representatives from 3 mice (n = 3). ADM: acellular dermal matrix.

The detergent-free method for decellularization of mouse skin and preparation of ADM utilizing Latrunculin B treatment, followed by hyper- and hypotonic-solution incubation and Benzonase^®^ Nuclease treatment has been described previously^45^. One to 3 days after seeding fibroblasts on ADM (8-mm diameter), it was washed with PBS three times, and transplantation of ADM and recellularized ADM was performed as previously reported^46^. Briefly, after induction of anesthesia and removing hair from the back skin of recipient wt-BALB/c mice, full-thickness excisional wounds, including the panniculus carnosus, were created in two different sites on the back using a 6-mm diameter punch device. Four wt-BALB/c mice per treatment group were used, and each mouse received either two ADMs or two fibroblast-recellularized ADMs. Grafts (8-mm diameter) were sutured on the tissue surrounding the wound bed (Fig. 1A). Immediately following surgery, OPSITE dressing (Smith & Nephew, Calgary, AB, Canada) was sprayed onto the graft site. Tegaderm film was applied over the graft site and then secured by a 2-cm width co-flex bandage.

### Histological Examinations

For histological evaluation of the harvested grafts, conventional H&E staining was done on 5-µm-thickness paraffin sections.

### *In Vivo* Depletion of Immune Cell Populations

Depletion of neutrophils and T-cell subsets was achieved by i.p. injection of 200 μg antibody (BioXCell, Lebanon, NH, USA) every 3 days, beginning 3 days prior to cell transplantation, using the following antibodies: anti-Ly6G (clone 1A8), anti-CD8α (clone 2.43), anti-CD4 (clone GK1.5), and anti-Vγ2 TCR (clone UC3-10A6). For macrophage and NK-cell depletion, 200 μg of Clodronate liposomes (Liposoma BV, Amsterdam, Netherlands) and anti-asialo GM1 (Cedarlane, Burlington, ON, Canada), respectively, was injected 24 hours before cell transplantation and 50 μg every 3 days after. Depletion of each cell type was confirmed by flow cytometry of peripheral blood mononuclear cell (PBMC) before injecting each new dose of antibodies (data not shown). Antibodies used for depletion were from different clones than those used in flow cytometry to confirm the depletion.

### Bioluminescence Imaging

For tracking cells *in vivo* and after transplantation, C57BL/6 fibroblasts in passage 2 and 3 cultured in 6-well plates, were transduced by a self-inactivating (SIN) lentiviral vector carrying a double fusion (DF) reporter gene of click beetle red (CBR) luciferase and enhanced green fluorescent protein (eGFP), driven by a constitutive ubiquitin promoter (pUB; Supplementary Fig. 1Aa). For transduction, lentiviral vector was added to 1 ml culture media and mixed with 8 µg/ml of polybrene (Sigma, H9268-5G) then added to the cells and centrifuged for 20 min at 1,200 × *g*. After centrifugation, cells were cultured for 2 days then flow sorted for eGFP expression. After sorting, fibroblasts were cultured for an additional two to three passages and transplanted by intradermal injection (2 × 10^5^ per spot) into the lateral side of dorsal skin of recipient mice (Supplementary Fig. 1B). Bioluminescence imaging (BLI) was first performed 6 hours (day 0) or 15 hours (day 1) after transplantation. After day 1, BLI was performed every other day. For BLI, mice were injected i.p. with 5 µg of D-Luciferin in PBS and imaged under isoflurane anesthesia 10 to 30 minutes later using an Ami-x system (Spectral Instruments Imaging, Tucson, Arizona, USA). Quantification of light emission (photons/second/cm^2^/sr) in regions of interest (ROIs) was performed using Aura software (Spectral Instruments Imaging). To account for the impact of initial differences in transplanted cell number on longitudinal experiments, the value obtained at each time point was normalized to the value for that same ROI on day 1.

### Isolation of Infiltrating Immune Cells at the Site of Cell Injection in the Skin

A 12-mm-diameter circle from the skin at the site of cell injection was harvested 5 days after transplantation and then minced using a blade. To achieve single-cell isolation, tissue samples were digested with Liberase™ enzyme blend (Sigma-Aldrich, Oakville, ON, Canada) at 37°C for 45 minutes in Hank’s Balanced Salt Solution with 250 r/min shaking. Digestion was stopped by adding RPMI media supplemented with 10% fetal bovine serum (FBS) and the cell suspension passed through a 40-µm pore size cell strainer and washed with same media.

### Flow Cytometry

The following fluorochrome-tagged antibodies (BD Pharmingen) were used for immune cell subset identification: (1) lymphocyte panel: APC-Cy7-CD3, PE-Cy7-CD19, BV605-CD45, V500-CD4, V450-CD8a, PE-CD69, PE-CF594-CD49b, BV650-CD335 (NKp46), and 7AAD (dead cell exclusion dye) and (2) myeloid panel: FITC-Gr-1, PE-Cy™7-CD11b, APC-Cy™7-Ly-6G, BV711-F4/80, and DAPI (dead cell exclusion dye). For the lymphocyte panel, we first gated 25,000 single viable cells (7AAD−) and then gated single CD45+ cell (7AAD−) and then evaluated activated CD4+ cells (CD19− CD3+ CD4+ CD69+), activated CD8+ cells (CD19− CD3+ CD8+ CD69+), NK cells (CD19-CD3− CD49b+ CD335+). For the myeloid panel, we first gated 25,000 single viable cells (DAPI−) and then gated on CD45+ cells and evaluated CD11b+Gr-1+ cells as monocytes, CD11b+ F4/80+ cells as macrophages, Gr-1+ Ly6G+ as neutrophils. Flow cytometry was performed on an LSR Fortessa flow cytometer (BD Biosciences, Mississauga, ON, Canada), and data were analyzed using FlowJo software (Tree Star Inc., Ashland, OR, USA).

### Real-Time Reverse Transcriptase-Polymerase Chain Reaction

Quantitative reverse transcriptase-polymerase chain reaction (qRT-PCR) was used to measure chemokine gene expression on days 1, 3, 6, and 12 after cell transplantation into allogeneic host. PBS-injected skin was used as a control to provide a baseline to account for changes in gene expression resulting from the trauma of needle insertion into the dermis-hypodermis. At each time point, mice were euthanized and the marked transplantation spot excised and transferred into the RNAlater solution (Life Technology, Toronto, ON, Canada). After 24 hours storage at 4°C, the solution was removed and tissue samples transferred to −80°C until RNA extraction. RNA extraction was performed using Trizol reagent (Invitrogen, Burlington, ON, Canada), according to the manufacturer’s protocol. cDNA was synthesized using 2 µg of total RNA and Maxima™ H minus cDNA synthesis master mix kit, with dsDNase (Life Technology). qPCR was performed in duplicate using the ViiA7 Instrument (Applied Biosystems, Mississauga, ON, Canada) and SYBR Green I fluorescence for detection. Primer sequences are listed in Supplementary Table 1. We used three mice per group, each with two transplantation spots at the lateral sides of the back skin for this experiment. Data were processed, and relative expression of the target genes was calculated following the method of DART-PCR (Data Analysis for Real-Time PCR)^47^.

### Statistical Methods

Numerical data are expressed as mean ± standard error. Statistical differences between the means for the different groups in BLI results were compared using two-way analysis of variance (ANOVA) and post hoc Bonferroni multiple comparison tests. For flowcytometry result, multiple unpaired *t*-tests was performed. Prism 9.0 software (GraphPad Software Inc., La Jolla, CA) was used for all statistical analysis. Specific n values for each experiment and the level of significance (*P* value) are listed in figure legends.

## Results

### ADM Recellularized With Allogeneic Fibroblasts Does Not Enhance Wound Healing

To investigate the outcome of repopulating allogeneic fibroblasts within the ADM as a wound coverage in full-thickness skin wound model in mice, we first examined cytocompatibility of ADM by analyzing attachment and viability of fibroblast after seeding them on ADM. Fibroblasts derived from skin of C57BL/6 mice were cultured for 24 hours on ADM surfaces, then analyzed using a Live/Dead staining kit. Fibroblasts adhered to ADM and remained viable over the first 24 hours (Supplementary Fig. 2A). The sustained viability of *in vitro* cultured fibroblasts on ADM was confirmed over 3 weeks by the absence of LDH in culture supernatant, except at day 16 which was equal to 500 dead cells in our assay control group, indicating that long time culture of cells on ADM does not induce significant cell death (Supplementary Fig. 2B).

ADM or recellularized ADM were transplanted into the full-thickness wound in wt-BALB/c mice (Fig. 1A). The presence of fibroblasts seeded on ADM was confirmed by analyzing recellularized ADM graft, 1 hour after transplantation (Fig. 1B). Three weeks after transplantation, histology results confirmed engraftment to surrounding host tissue with complete epithelialization, and the presence of host cells within the grafts [Fig. 1C(i, ii)]. Similar results were observed with recellularized ADM grafts [Fig. 1C(iii, iv)]. There was, however, no difference between experimental groups in terms of time required for healing and the re-epithelialization of the graft. In the recellularized ADM group, high cellularity can be seen at the interface of graft/host tissue (arrow heads) [Fig. 1C(iv)]. Such cellularity was not observed in the ADM (data not shown). Staining of the graft for pan-leukocyte marker, CD45, revealed that majority of the cells in the high cellularity regions are CD45+ cells [Fig. 1D(i, ii)].

### Immune-Mediated Rejection of Transplanted Allogeneic Fibroblasts

**Figure 2.**
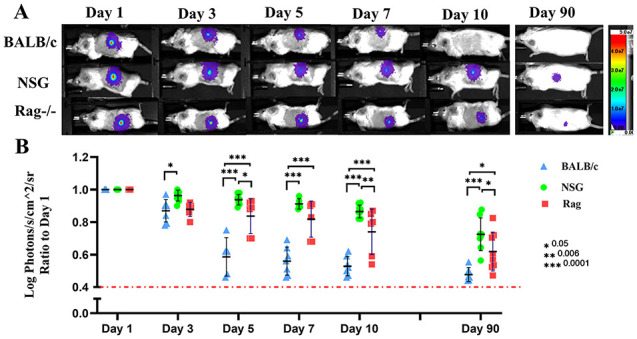
BLI of transplanted fibroblasts into wild-type BALB/c mice as an allogeneic recipient and into immune-deficient RAG−/− and NSG mice (A, B). (n = 5–8) and *P* values are *0.05, **0.005, and ***0.0001. Measurement of light emission (Log photons/second/cm^2^/sr) in regions of interest (ROIs) was used to quantify the bioluminescent signals. The red dashed line represents the background signal level detected from skin. BLI: bioluminescence imaging; NSG: γ chain–deficient NOD-SCID.

From the transplantation experiments, it is unclear whether fibroblasts are retained in ADM over time but do not contribute in healing process, migrate from the graft, or die as a result of immune rejection. To trace the transplanted cells longitudinally, we transduced donor fibroblasts with a dual luciferase-EGFP reporter construct (Supplementary Fig. 1A.a–e). To reduce the effect of variables within the wound microenvironment on transplanted cells, luciferase-EGFP labeled allogeneic fibroblasts were injected intradermally into immune-competent wild-type and immune-deficient RAG−/− BALB/c mice and NSG mice and tracked by BLI (Fig. 2). As dermis/hypodermis is a well-vascularized tissue, intradermal injection of cells minimizes the potential role of delayed angiogenesis and oxygen/nutrition deprivation as causes of fibroblast loss. In wt-BALB/c mice, the majority of transplanted cells was not detectable within 5 to 7 days of injection and was rejected in all recipients by 10 days. In contrast, the fibroblast-derived BLI signal was barely diminished over the first 10 days in lymphocyte-deficient NSG mice. Despite a decrease in signal, fibroblasts could still be detected at the injection site after 90 days in NSG mice, confirming the survival capacity of the fibroblasts and indicating that loss of signal is unlikely to be the result of bulk migration of fibroblasts out of the original site. Compared to NSG mice, the T-cell- and B-cell-deficient Rag−/− mice showed greater cell loss by day 5 after transplantation (*P* ≤ 0.05). However, survival of fibroblasts was significantly prolonged compared to wt-BALB/c mice, with cells persisting to day 10 in most recipients. Fibroblasts survived to day 90 in a few of Rag−/− recipients, although at a significantly lower number than in NSG mice (*P* ≤ 0.05). Overall, these findings implicate both the innate and adaptive arms of the immune response in the loss of allogeneic fibroblasts for the injection site.

### Inflammation at the Fibroblast Injection Site

To identify the immune cells recruited to the fibroblast injection site at the time of rejection, at day 5 after transplantation, we isolated cells from harvested tissues and analyzed them by flow cytometry.

**Figure 3.**
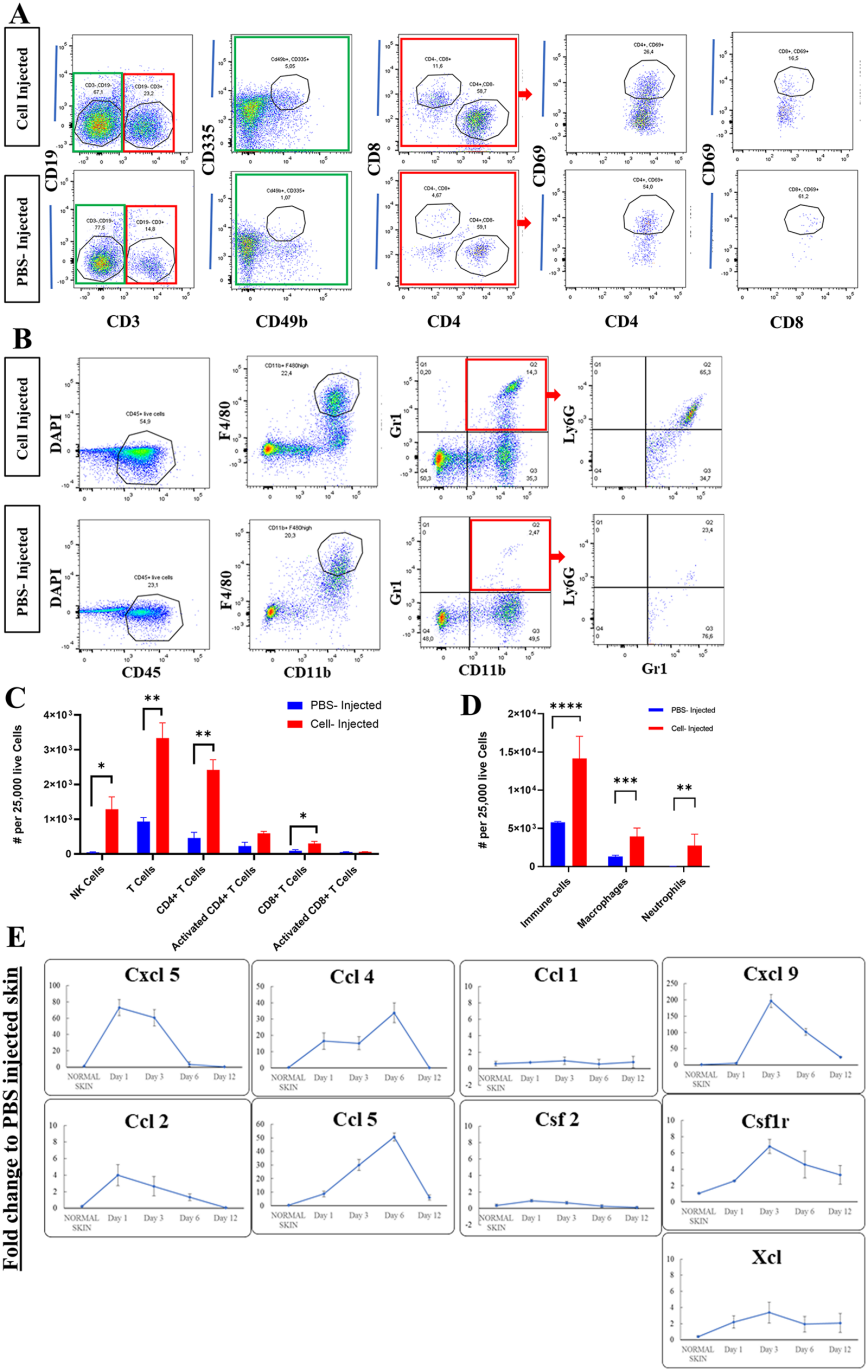
Infiltration of T-cells (CD3+ CD4+ and CD3+ CD8+) and NK cells (CD3− CD49b+ CD335+), *P* values are *0.029, **0.004, ***0.001 (A, C) and macrophage (CD11b+ F4/80+) and neutrophils (CD11b+ Gr1+ Ly6G+) to the site of fibroblasts transplantation at day 5. *P* values are **0.007, ***0.001, ****0.0008 (B, D). Proinflammatory chemokines/chemokine receptors showed elevated level of gene expression during allogeneic fibroblasts rejection (n = 3) (E). PBS: phosphate-buffered saline; DAPI: 4′, 6-diamidino-2-phenylindole.

We found that a large number of host myeloid cells, mainly macrophages, and neutrophils, infiltrated into fibroblast transplant sites compared to vehicle (PBS)-injected sites (Fig. 3B, D). Interestingly, there was a comparably high number of CD4+ T-cells in the site of cell transplantation, but few CD8+ cells. Using CD69 as an early activation marker for T-cells, there was no significant difference in the number of activated CD4+ and activated CD8+ T-cells compared to PBS-injected control group (*P* = 0.129 and *P* = 0.657, respectively; Fig. 3A, C). We also found that infiltration of NK cells (CD3− CD49b+ CD335+) in allogeneic cell–injected samples was higher than PBS-injected samples (Fig. 3A, C).

To assess which chemokine/chemokine receptor networks are directing immune cell migration to the site of transplant, we compared gene expression at injection site between cell-transplanted allogeneic hosts and PBS-injected controls (Fig. 3E). Within 24 hours of transplantation, elevated levels of Cxcl5, Ccl2, Ccl4, and Ccl5 gene expressions were detectable in cell recipients. Gene expression level for Ccl4 and Ccl5 continued to increase up to Day 6. The second set of genes, including Cxcl9, Xcl, and Csf1r, did not show any upregulation on the first-day postinjection but were increased on days 3 and 6. Other genes, including Ccl1 and Csf2, did not show any changes between test and control groups. The high level of proinflammatory chemokine/chemokine receptor genes expression and the infiltration of myeloid cells, mainly macrophages and neutrophils detected at the injection site within the first week after transplantation, were indicative of an early inflammatory response mediated by innate immune cells.

### Neutrophil- and Macrophage-Depletion Can Delay Allogeneic Fibroblast Rejection in the First Week of Transplantation

**Figure 4.**
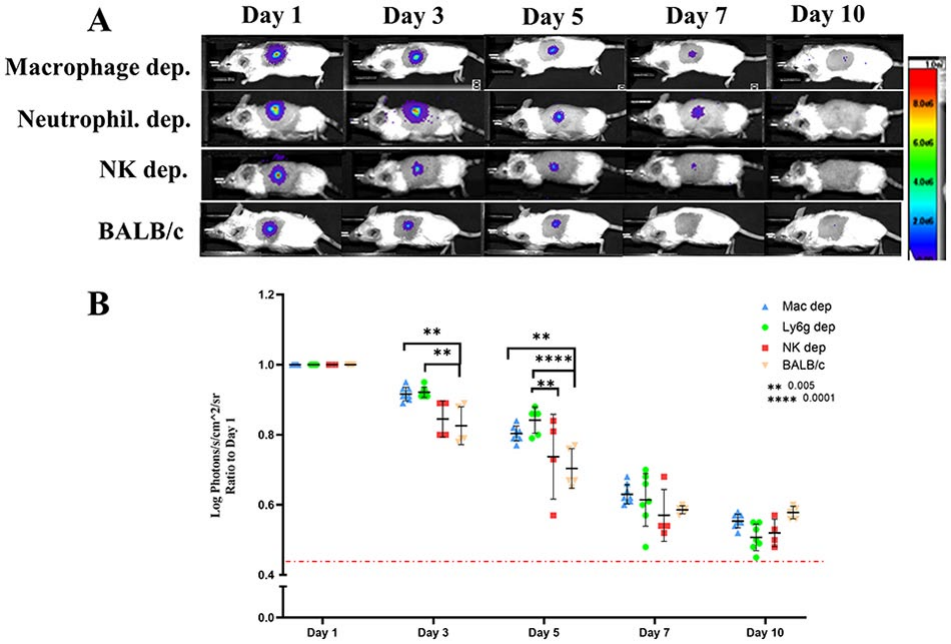
Depletion of macrophages and neutrophils delayed immune rejection of fibroblasts in first week after transplantation but could not prevent it. NK depletion did not have significant impact on preventing the rejection. (n = 4–6) and *P* values are **0.005, ****0.0001 (A, B). Injection of clodronate-loaded liposomes, Ly6G antibody, or asialo-GM1 antibody (ASGM-1) to wt-BALB/c mice used to deplete macrophage, neutrophils, and NK cells, respectively. Depletion treatment was started 3 days before cell transplantation and continued every 3 days up to 9 days posttransplantation. Measurement of light emission (Log photons/second/cm^2^/sr) in regions of interest (ROIs) was used to quantify the bioluminescent signals. The red dashed line represents the background signal level detected from skin. Wt: wild-type; NK: n﻿atural k﻿iller cells.

The BLI signal differences in between NSG and Rag−/− mice, together with the observed immune cell infiltration, strongly implicated innate responses in the early loss of transplanted fibroblasts. To investigate this role, we administered clodronate-loaded liposomes, Ly6G antibody, or asialo-GM1 antibody (ASGM-1) to wt-BALB/c mice to deplete macrophage, neutrophils, and NK cells, respectively. Depletion of neutrophils and macrophage significantly reduced the rate of rejection in the first 5 days posttransplantation in comparison with non-depleted wt-BALB/c; however, such depletion did not prevent later rejection (Fig. 4A, B). Notably, we could not achieve complete depletion of macrophage either in spleen or at the site of cell injection in the skin (Supplementary Fig. 3) as administration of clodronate liposome was toxic to the mice in higher doses. Despite evidence of their recruitment to the injection site, NK cell depletion (Supplementary Fig. 4) did not prolong the survival of transplanted cells (Fig. 4A, B).

### Depletion of Both CD4+ and CD8+ Cells Is Necessary for Long-Term Survival of Fibroblasts

**Figure 5.**
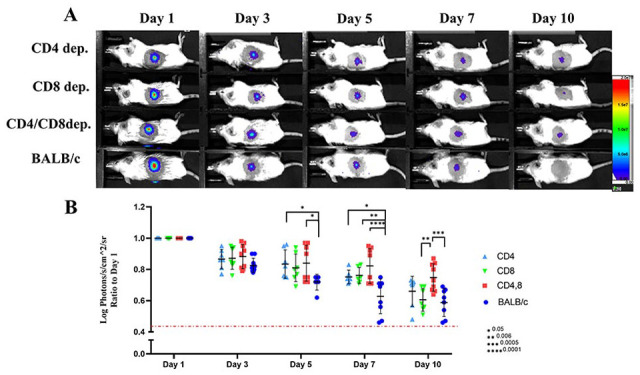
Simultaneous depletion of CD4+ and CD8+ cells could partially prevent rejection of fibroblasts (n = 6–8) and *P* values are *0.05, **0.006, ***0.0005, ****0.0001 (A, B). Wt-BALB/c mice were injected with antibodies to CD4, CD8, or both, starting 3 days before cell transplantation and continued every 3 days up to 9 days posttransplantation. Measurement of light emission (Log photons/second/cm^2^/sr) in regions of interest (ROIs) was used to quantify the bioluminescent signals. The red dashed line represents the background signal level detected from skin. Wt: wild-type.

To assess the role of T-cell subsets in the rejection of fibroblasts, wt-BALB/c mice were injected with antibodies to CD4, CD8, or both, starting three days before cell transplantation and continued every 3 days up to 9 days posttransplantation. T-cell depletion from peripheral blood samples was monitored by flow cytometry before each new dose of antibodies (Supplementary Fig. 5). While single depletion of CD4+ or CD8+ cells somewhat delayed immune rejection, only double-depletion significantly improved fibroblast survival at day 10 (Fig. 5A, B).

**Figure 6.**
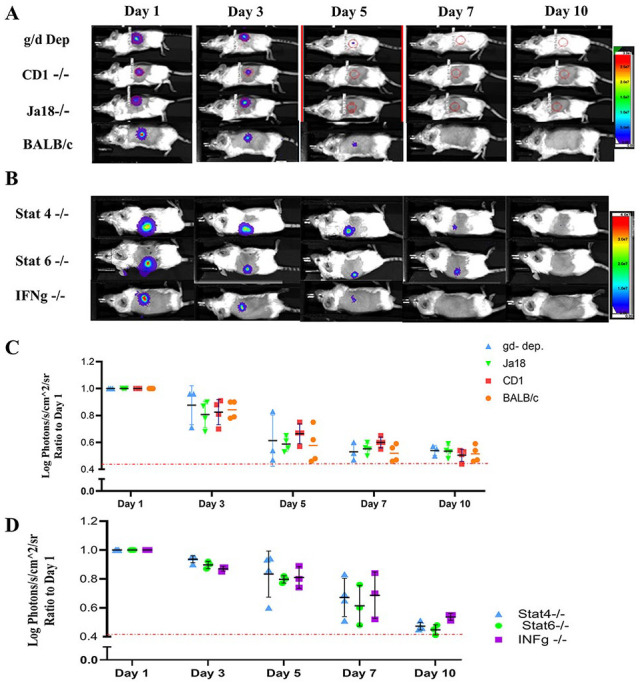
γδ T-cells and NKT cells were not involved in immune rejection of fibroblasts. γδ T-cells were depleted from the recipient mice by injecting 200 μg of anti-Vγ2 TCR antibody every 3 days, beginning 3 days prior to cell transplantation and continued up to 6 days posttransplantation. Ja18 knockout (Ja18−/−, type-1 NKT cell deficient) and CD1d knockout (CD1d−/−, pan NKT cell deficient) mice were used to evaluate the role of NLT cells (n = 3–5) (A,C). Inhibition of Th1 and Th2 pathways cannot prevent immune rejection of fibroblasts. Stat4 and interferon-gamma (IFN-gamma) knockout mice are impaired in Th1 pathway and Stat6 knockout mice in Th2 pathway (B, D) (n = 3–4). Measurement of light emission (Log photons/second/cm^2^/sr) in regions of interest (ROIs) was used to quantify the bioluminescent signals. The red dashed line represents the background signal level detected from skin. NKT: natural killer T; TCR: T cell receptor.

As CD4 and CD8 markers are expressed by multiple T-cell subsets, we next evaluated the contribution of innate T-cells to early and late fibroblast rejection. To determine the role of natural killer (NK) T-cells, we injected allogeneic fibroblasts intradermally into Ja18 knockout (Ja18−/−, type 1 NKT cell deficient) and CD1d knockout (CD1d−/−, pan NKT cell deficient) mice. The role of γδ T-cells in this allogeneic rejection was also investigated by injecting allogeneic fibroblasts to γδ T-cell-depleted mice. In all three settings, fibroblasts were rejected with similar kinetics to that observed in wt-BALB/c recipients (Fig. 6A, C), suggesting that neither NKT nor γδ T-cells were involved.

Last, as our T-cell depletion experiments indicated that adaptive T-cells were significant contributors to fibroblast rejection, we next evaluated need for polarization of the T-cell response. To examine the involvement of Th1 and Th2 signaling pathways, we transplanted the allogeneic fibroblasts into Stat4, interferon-gamma (IFN-gamma), and Stat6 knockout mice. Stat4 and IFN-gamma knockout mice are impaired in Th1 pathway and Stat6 knockout mice in Th2 pathway. No extension of fibroblast survival was achieved in either setting (Fig. 6B, D).

## Discussion

Fibroblast-based tissue engineering products have been studied for the regeneration of different tissues, and some products are commercially available or in clinical trials for skin regeneration^7^. As an off-the-shelf product, allogeneic fibroblasts would represent the optimal choice to include in engineered tissue. However, the viability, persistence, and overall efficacy of transplanted allogeneic cells in immunocompetent hosts have been controversial^24,48–53^. In most of these experimental and clinical studies, the immunogenicity of fibroblasts, which can exert both proinflammatory and immunosuppressive activities depending on the microenvironmental conditions, has been largely unstudied. As ADM with allogeneic fibroblasts did not enhance wound healing in our study, we investigated the impact of recipient immune response on the long-term fate of allogeneic fibroblasts transplanted in mouse skin.

In previous studies, limitations in the ability to follow transplanted cells may have contributed to the conflicting reports regarding allogeneic fibroblast fate. To overcome this, we injected luciferase-expressing fibroblasts intradermally to circumvent the potential deleterious effect of delayed vascularization of grafted ADM, and used using BLI to detect any cell migration into surrounding tissues and to evaluate extended viability. Using this approach to evaluate engraftment over a 90-day experiment, comparison of transplanted cell survival in wild-type and immune-deficient mice revealed endogenous immune activity to be the primary modulator of transplanted allogeneic fibroblast persistence.

Our results indicate that innate immune activity is responsible for an early depletion of transplanted fibroblasts, which is consistent with the elevated level of proinflammatory chemokines detected at the injection site within 24 hours and the infiltration of a large number of myeloid cells, mainly macrophages and neutrophils, by day 5 after transplantation. Neutrophils have been reported to recruit to the graft site after transplantation^54^ and are important in wound healing to remove foreign material, bacteria, dead cells, and degraded ECM components by phagocytosis^55^. However, excessive infiltration by neutrophils is responsible for developing chronic inflammation that can lead to excessive tissue damage. Macrophages are recruited to wound areas 24 to 36 hours after injury in response to proinflammatory cytokines such as interleukin (IL)-1 and IL-6. By producing reactive oxygen species and proteolytic enzymes, they can cause injury to the tissue^54^. Macrophages have been shown to contribute to acute and chronic allograft and stem cell–derived cell rejection^56,57^. The delay in the fibroblast rejection achieved by depletion of macrophage and neutrophils in our study indicates the possible contribution of these cell activities in immune rejection of fibroblasts. NK cells were also detectable at the transplant site and rejection of allogeneic cells by NK cells via missing self-recognition has been reported in other studies^58,59^. In contrast to neutrophil and macrophage depletion, however, loss of NK cell activity did not prevent the rejection of cells.

As our cell culture and transplantations were performed under sterile conditions, it is unlikely that the observed innate immune cell responses were attributable to microbial antigens, implicating the allogeneic fibroblasts themselves in the recruitment of innate immune cells. There is evidence that fibroblasts provide proinflammatory mediators and may contribute to the recruitment of inflammatory cells, a function well described for endothelial cells^32,33^. It has been shown that cardiac fibroblasts can express intercellular adhesion molecule (ICAM) and vascular cell adhesion molecule (VCAM) on their surface in response to proinflammatory stimuli, which promote leukocyte sequestration and transmigration to the site of injury^60^. This function has been supported by a report of adhesion of neutrophils and B lymphocytes to cardiac fibroblasts^61^. A proinflammatory response by fibroblasts in the context of microorganism infection can induce an innate immune response against microorganisms mediated by toll-like receptors (TLRs), antimicrobial peptides, proinflammatory cytokines and chemokines, and growth factors^34,62,63^. There is a growing body of evidence that monocytes and macrophages distinguish between self and allogeneic non-self^58,64–67^. It has been shown that polymorphism in the gene encoding signal regulatory protein α (SIRP-α), which binds to the monomorphic receptor, CD47, is essential for the monocyte allorecognition response in the marrow plug transplantation model^68^. Whether the SIRP-α–CD47 interaction is also playing a role in macrophage non-self-recognition and immune response against allogeneic fibroblasts requires further investigation.

Infiltration of CD4+ and CD8+ T-cells into the cell injection site was observed 5 days after transplantation. However, there was no significant change in expression of CD69 on these two subtypes of T-cells between PBS and cell-injected groups. We used CD69 as an early activation marker for T-cells, but it can also be expressed on tissue resident memory T-cells^69–71^. Our flow cytometry panel could not distinguish between these cell types, and as we did not see any significant difference in CD69+ cell between PBS and cell-injected groups, we did not further investigate these T-cell populations. *In vitro* studies have shown that fibroblasts can mediate suppression of T-cell proliferation by a variety of mechanisms^35–40,72,73^. However, in our study, CD4+ cell depletion delayed fibroblast rejection, and simultaneous depletion of CD4+, and CD8+ cells prevented rejection to a similar degree as observed in Rag1-knockout mice. These results indicate that effector T-cell mechanisms were active and contributed significantly to loss of transplanted fibroblasts. Our T-cell subset depletion experiments indicate that the more innate-like T-cells (NKT and γδ T-cells) were unlikely to play a major role in fibroblast rejection.

## Supplemental Material

sj-docx-1-cll-10.1177_09636897221113803 – Supplemental material for Inflammatory Immune Responses Trigger Rejection of Allogeneic Fibroblasts Transplanted into Mouse Skin Click here for additional data file. Supplemental material, sj-docx-1-cll-10.1177_09636897221113803 for Inflammatory Immune Responses Trigger Rejection of Allogeneic Fibroblasts Transplanted into Mouse Skin by Ali Farrokhi, MohammadReza Rahavi, Sumin Jo, Reza Jalili, C. James Lim, Aziz Ghahsary and Gregor S. D. Reid in Cell Transplantation
