# Supplementary material for: Inflammatory Immune Responses Trigger Rejection of Allogeneic Fibroblasts Transplanted into Mouse Skin
Source: Cell Transplant. 2022 Jul 30;31:09636897221113803. doi: 10.1177/09636897221113803 (PMC9340901; doi:10.1177/09636897221113803)
Supplement: sj-docx-1-cll-10.1177_09636897221113803 – Supplemental material for Inflammatory Immune Responses Trigger Rejection of Allogeneic Fibroblasts Transplanted into Mouse Skin [file sj-docx-1-cll-10.1177_09636897221113803.docx]

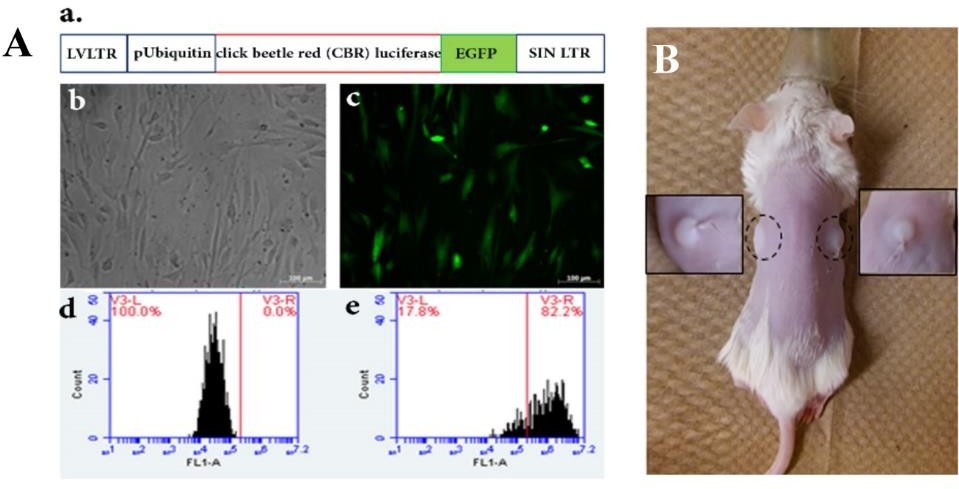


**Supplementary Figure 1; A)** Dual reporter Luciferase-EGFP lentivirus transduction of B6 fibroblasts. **a)** For tracking cells *in vivo* and after transplantation, C57BL/6 fibroblasts in passage 2-3 cultured in 6- well plates, were transduced by a self-inactivating (SIN) lentiviral vector carrying a double fusion (DF) reporter gene of click beetle red (CBR) luciferase and enhanced green fluorescent protein (eGFP), driven by a constitutive ubiquitin promoter (pUB). **b,c)** brightfield and fluorescent images from transduced cells. **d,e)** flowcytometry detection of GFP positive cells in non-transduced (d) and transduced (e) cells. non-transduced cells were used as a control for gating. **B)** Intradermal injection of 2×105 sorted cells into the lateral side of dorsal skin of recipient mice.


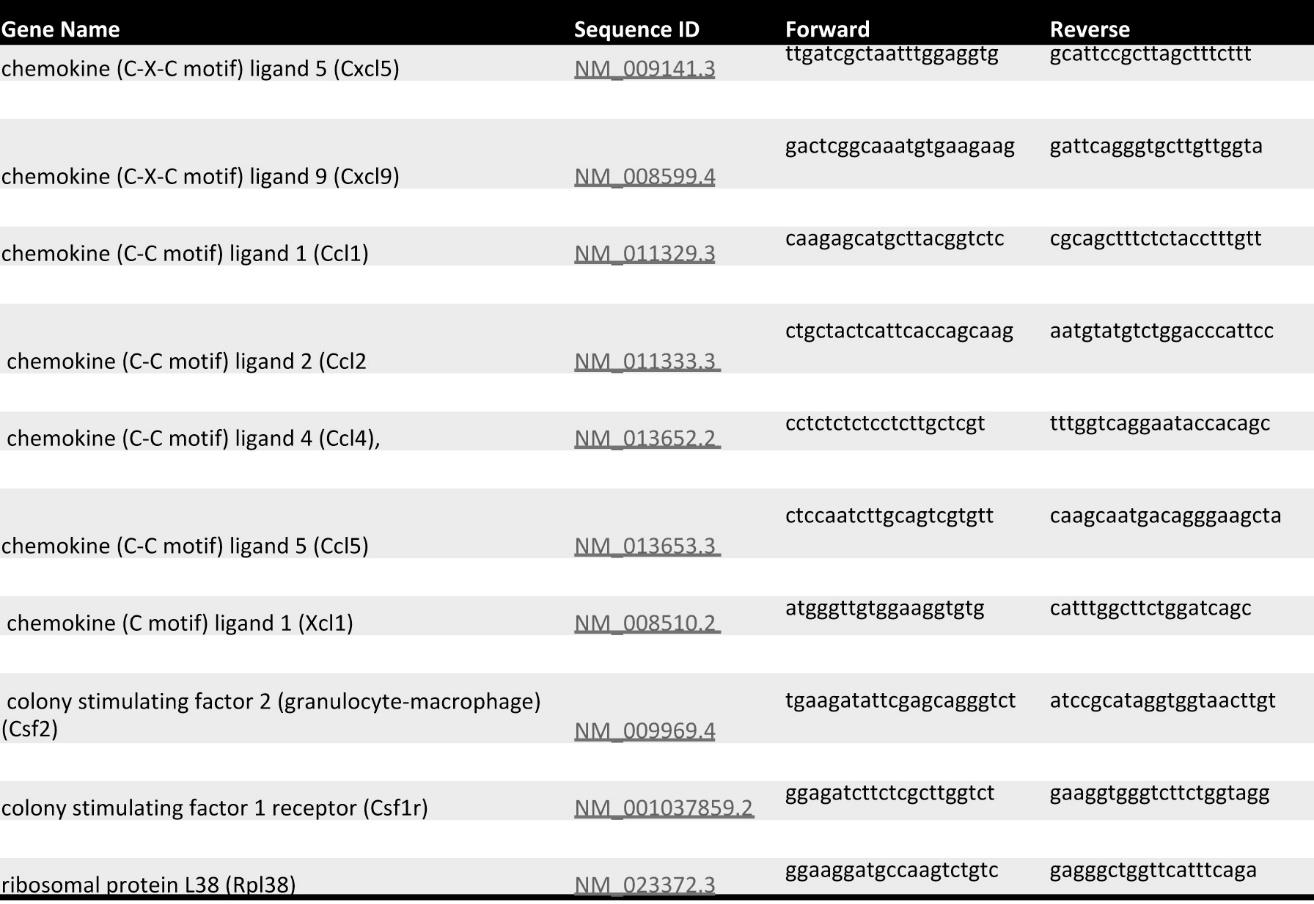


**Supplementary Table 1:** Primers used for analyzing chemokines/chemokine receptors gene expression in the site of cells transplantation.


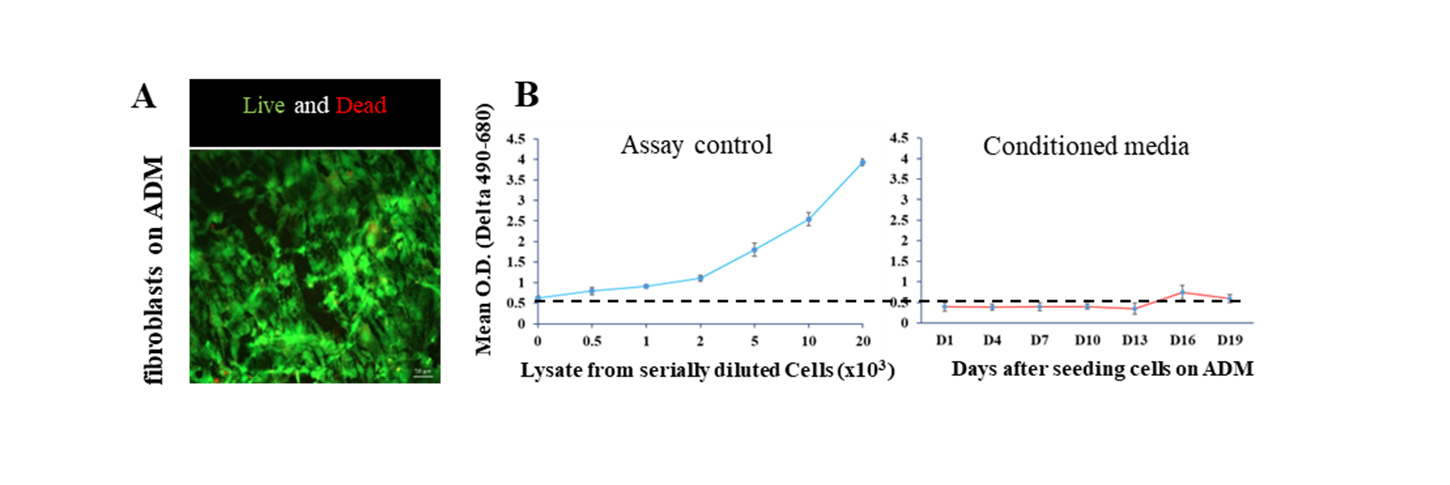


**Supplementary Figure 2;** Cytocompatibility of ADM. **A)** Cell adherence and viability was assessed 24 hours after seeding, using a Live/Dead toxicity staining. **B)** LDH assay was performed on conditioned media collected every three days for three weeks from fibroblasts cultured on ADM and showed high viability rate (n=4). Lysate from serially diluted *in vitro* cultured fibroblasts (0.5-20 x10^3^ cells) used as assay control to detect LDH. Dotted line represents the baseline for detecting LDH which is equal to the OD of solution without any cells.


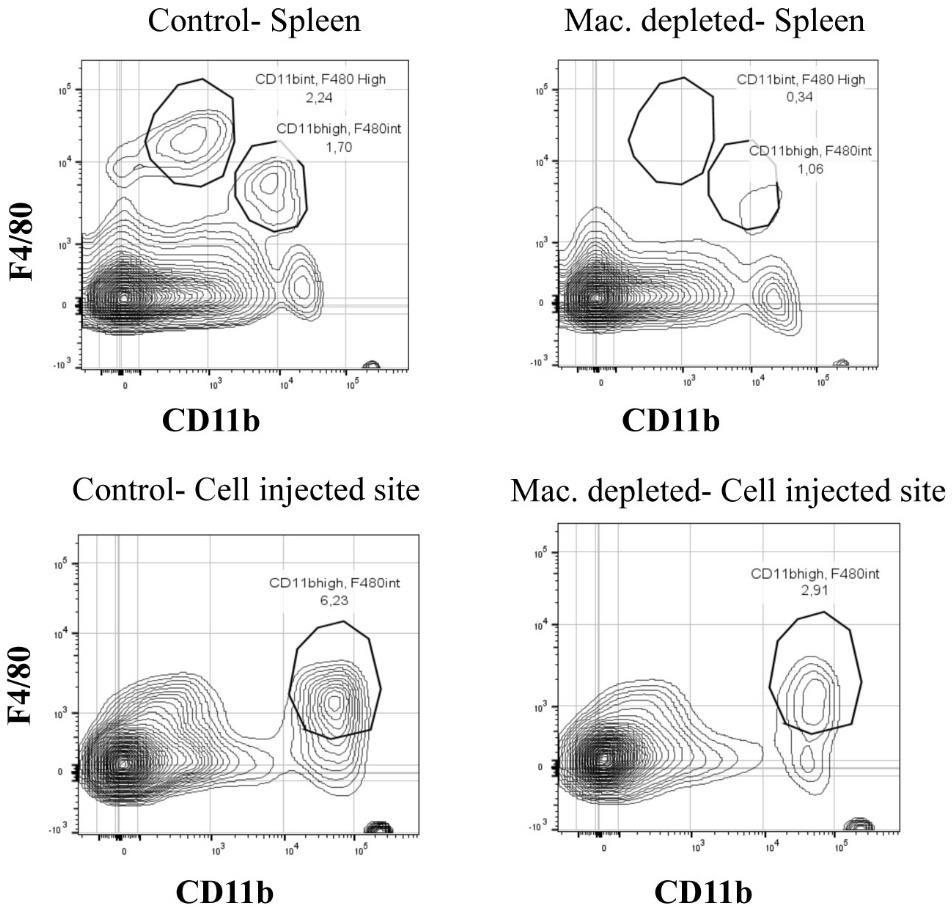


**Supplementary Figure 3.** The efficiency of macrophages depletion by clodronate liposomes injection was monitored using flow cytometry. Six days after transplantation, spleen and cell transplanted spot on the skin of clodronate liposomes treated (Mac. depleted) and untreated (control) mice were removed.

Then after homogenization, preparation of single cell suspension, and staining for flowcytometry, macrophages were identified as CD11b+F4/80+ cells, after exclusion of dead cells.


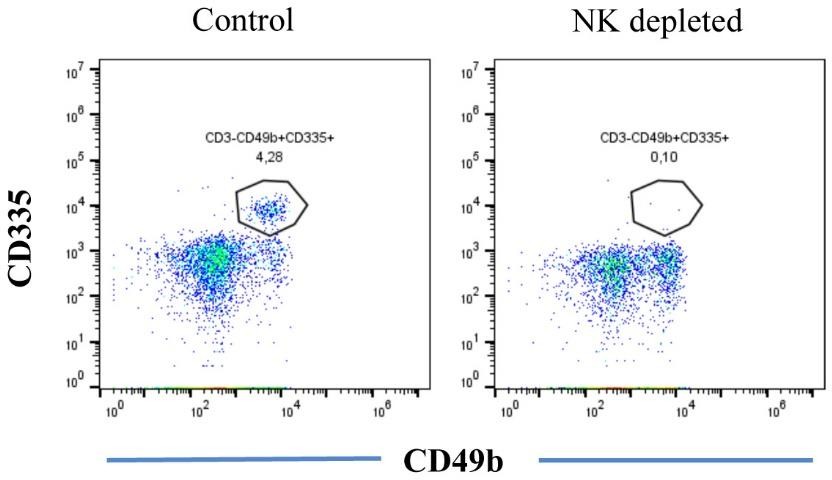


**Supplementary Figure 4.** The efficiency of NK depletion by anti-asialo GM1 injection was monitored using flow cytometry on spleen, 6 days after transplantation.


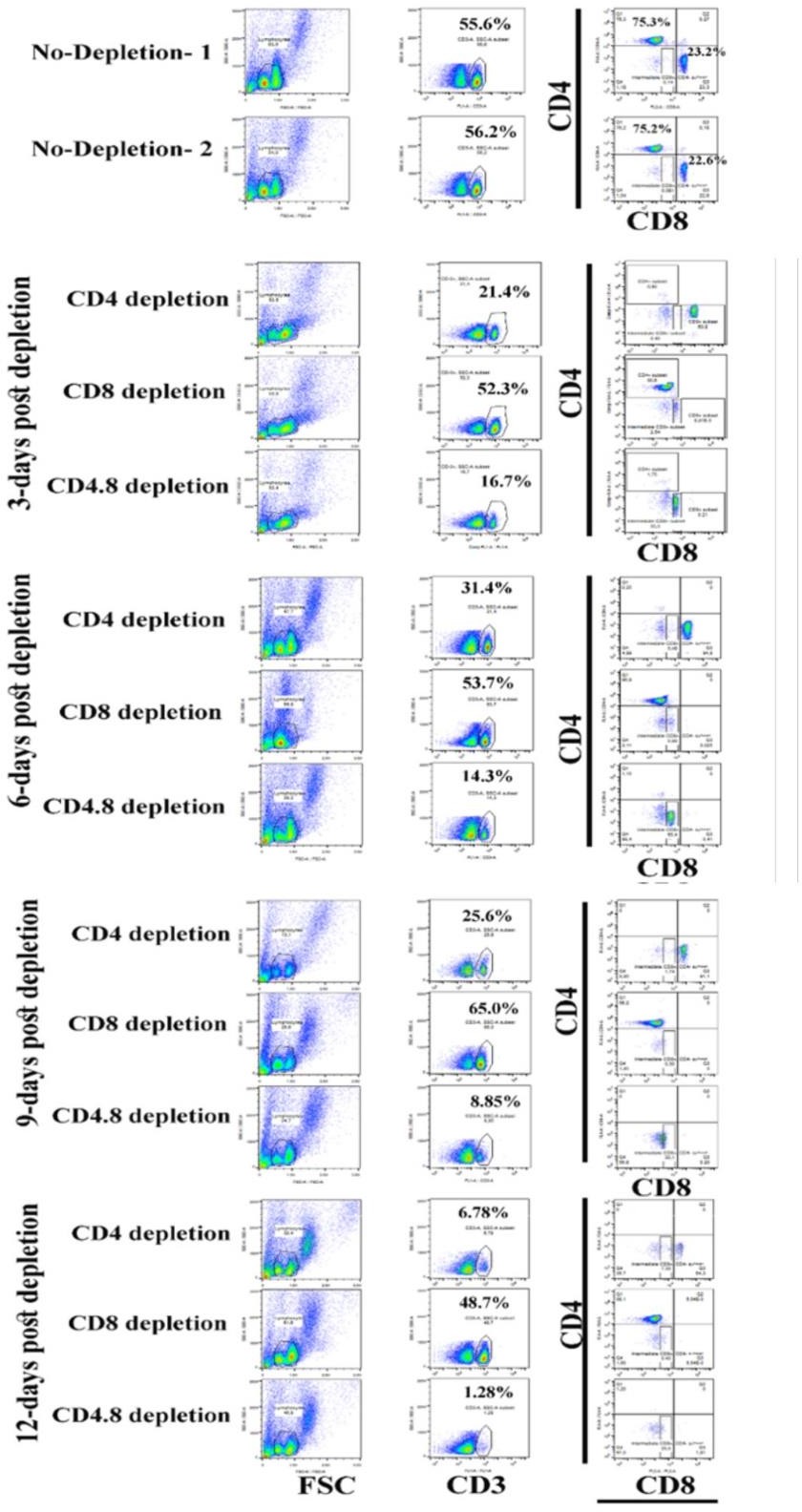


**Supplementary Figure 5;** The efficiency of T cell depletion by each CD4 and CD8 antibodies or combination of both antibodies was monitored using flow cytometry on peripheral blood samples every three days before injecting a new dose of antibodies.
